# Bioprinting of mesenchymal stem cells in low concentration gelatin methacryloyl/alginate blends without ionic crosslinking of alginate

**DOI:** 10.1038/s41598-025-90389-2

**Published:** 2025-02-24

**Authors:** Masoumeh Jahani Kadousaraei, Shuntaro Yamada, Mehmet Serhat Aydin, Ahmad Rashad, Noemi Molina Cabeza, Samih Mohamed-Ahmed, Cecilie G. Gjerde, Michael Malkoch, Kamal Mustafa

**Affiliations:** 1 Tissue Engineering Group, Center of Translational Oral Research, Department of Clinical Dentistry, University of Bergen, Bergen, Norway; 2 Bioengineering Graduate Program, Aerospace and Mechanical Engineering, University of Notre Dame, Notre Dame, IN 46556 USA; 3 Division of Coating Technology and Division of Biocomposites, Department of Fiber and Polymer Technology, School of Chemical Science and Engineering, KTH Royal Institute of Technology, Stockholm, Sweden

**Keywords:** 3D bioprinting, Calcium chloride, Photocrosslinking, Interpenetrating network, Mesenchymal stem cells, Tissue engineering, Mesenchymal stem cells, Stem-cell research

## Abstract

Bioprinting allows for the fabrication of tissue-like constructs by precise architecture and positioning of the bioactive hydrogels with living cells. This study was performed to determine the effect of very low concentrations of alginate (0.1, 0.3, and 0.5% w/v) on bioprinting of bone marrow mesenchymal stem cells (BMSC) in gelatin methacryloyl (GelMA; 5% w/v)/alginate blend. Furthermore, while GelMA was photocrosslinked in all bioprinted constructs, the effect of crosslinking alginate with calcium chloride on the physical and biological characteristics of the constructs was investigated. The inclusion of low-concentration alginate improved the viscosity and printability of the formulation as well as the compressive modulus of the hydrogels, particularly when ionically crosslinked with calcium chloride, compared with the group in that alginate was not crosslinked. However, the stability and degradability of 3D printed scaffolds that were only photocrosslinked were comparable to those that were additionally crosslinked with calcium chloride. Noteworthily, ionic crosslinking of alginate deteriorated the viability of BMSC. Morphology and growth of BMSC were improved by adding a low alginate concentration; however, ionic crosslinking of alginate affected these factors adversely. The findings of this study underscore the significance of carefully evaluating the crosslinking strategy used in conjunction with cell-laden GelMA/alginate hydrogel to achieve balanced physical and biological properties as well as less complicated post-bioprinting processing.

## Introduction

In the field of tissue engineering, three-dimensional (3D) bioprinting has emerged as a promising approach for the precise biofabrication of patient-specific shaped constructs^1–3^. Of particular interest is the process of extrusion-based bioprinting (EBB), offering complete control over the design of the scaffolds, with a layer-by-layer approach, to recreate 3D macroporous structures^3,4^. The bioinks used in EEB often consist of natural polymers (e.g., collagen, gelatin, alginate), biological factors, and living cells^5,6^. When selecting biomaterials for EEB-based bioinks, the main criteria are resolution, structural stability, degradation rate, optimum rheological properties, and viscoelasticity^7–9^. There is equal emphasis on optimal biological properties, including the viability and functionality of encapsulated cells^10,11^. The cellular responses depend not only on biomaterial-ink properties but also on printing parameters such as needle (nozzle) size, temperature, pressure, and shear stress^1,11,12^.

Among the wide range of potential biomaterials, gelatin methacryloyl (GelMA), prepared by introducing methacrylic anhydride (MA) on reactive amine and hydroxyl groups of the amino acid residues of gelatin structure to make it photocrosslinkable, has shown outstanding potential in EEB-based 3D bioprinting of cells^13–15^. Photocrosslinking is a process in which light activates a photoinitiator to generate free radicals that attack the double-bonds in the methacryloyl groups, initiating polymerization and forming crosslinks between polymer chains, thereby converting a hydrogel precursor into a solid, 3D network^16^. Among important features are its biodegradability, ideal biocompatibility, and its abundant natural mammalian cell-adhesive recognition sites such as RGD (Arginine-Glycine-Aspartic acid)^17,18^. However, it is reported that low concentrations of GelMA require the incorporation of other polymer components, such as alginate—a linear polysaccharide consisting of β-D-mannuronic acid (M) and α-l-guluronic acid (G) monomers^19^—to achieve enhanced viscosity, material extrudability, and structural stability^20,21^. The optimum shape fidelity in bioprinting using bioink formulations is usually attained by elevating polymer concentrations^20,22^. However, high-density hydrogel networks can generate substantial shear forces on cells during the bioprinting process, potentially compromising cell viability^23^. In addition, to enhance the mechanical properties of the constructs, carboxyl groups of alginate are commonly crosslinked ionically with divalent cations such as calcium ions (i.e., calcium chloride; CaCl_2_)^20,24^. The elastic moduli of calcium ion-crosslinked alginate hydrogels are reported within the range of 1 to 100 kPa, with variations depending on the concentration of calcium ions and the specific composition of the alginate chains^25,26^. However, alginate hydrogels formed through ionic crosslinking exhibit a compact network structure, limited biodegradability in mammalian system, and high stiffness, which markedly impede cell migration and spreading^27,28^. Furthermore, disruption of cells’ internal calcium balance, often due to excessive calcium influx, can damage the cellular mitochondria and cytoskeleton and initiate a programmed cell death caused by osmotic stress^29–31^. Despite the reported cellular damage attributed to calcium ions, extensive studies on GelMA/alginate hydrogels continue to employ calcium-mediated crosslinking of alginate^32–35^. Based on the available literature, there exists a limited investigation of non-crosslinked alginate in bioprinted GelMA/alginate hydrogel composites with human bone marrow mesenchymal stem cells (BMSC), leaving the potential of non-crosslinked alginate in terms of mechanical properties, degradation profiles, and cell behavior largely unexplored.

In this study, we aimed to examine the impact of low alginate concentrations (below 0.5% w/v) in GelMA polymer on the physical properties, including rheological properties, printability, and shape fidelity of the formulations. Furthermore, we aimed to determine whether the alginate-containing bioinks without ionic crosslinking of alginate, with only photocrosslinked GelMA, provide proper mechanical properties, structural integrity, stability, and biocompatibility, compared to the scaffolds that alginate is ionically crosslinked. We hypothesized that the entanglement of alginate chains in a crosslinked GelMA matrix, without external ionic-crosslinking of alginate, would provide proper stability and environment for cell functionality^36^. This study provides insights into the effect of introducing low concentrations of alginate as a sacrificial material in GelMA-based bioinks on the physical and biological behavior of bioprinted scaffolds to develop a less complicated post-bioprinting processing.

## Materials and methods

### Inks and hydrogels preparation

GelMA/alginate solutions were prepared by a mixture of 5% (w/v) GMP-grade GelMA (X-pure® GelMA 160P60; MW 160 kDa with 60% degree of methacrylation; Rousselot, Belgium) with different and low concentrations of alginate (High molecular weight alginate; Nova-Matrix, Norway) ranging from 0.1%, 0.3%, to 0.5% (w/v) in Minimum Essential Medium α (MEMα; 22571-020; ThermoFisher Scientific, USA) solution supplemented with 1% penicillin/streptomycin (P/S; SV30010; cytiva HyClone, 10,000 U/mL; ThermoFisher Scientific). Lithium-Phenyl-2,4,6-trimethylbenzoyl phosphinate (LAP, 0.25% w/v; Tokyo Chemical Industry Co., Ltd., Japan) was used as a photoinitiator to photocrosslink GelMA polymers. Groups of GelMA only, and GelMA with different alginate content of 0.1, 0.3, and 0.5% w/v, were referred to as GelMA, GelMA/Alg 0.1, GelMA/Alg 0.3, and GelMA/Alg 0.5, respectively. GelMA polymers were photocrosslinked in all hydrogel groups (single-crosslinked) with a Bluephase® PowerCure Dental lamp (Ivoclar Vivadent; Liechtenstein) with 1200 mW/cm^2^ intensity visible light for two cycles of 30 s. Alginate crosslinking was divided into two groups with and without ionic crosslinking with 10 min incubation in 100 mM CaCl_2_ (Calcium chloride dihydrate, Sigma-Aldrich, USA). In total, these formulations and different crosslinking conditions resulted in seven hydrogel blends. A summary of groups is provided in Table 1. The hydrogel groups that were only photocrosslinked are indicated with (−) and the groups that had both photocrosslinking and ionic crosslinking are indicated with (+). The design of the experiment is presented in Fig. 1. First, GelMA was dissolved in MEMα-P/S at 40 °C under stirring condition for 30 min. Alginate powder was then added to the GelMA solution and mixed for 1 h at 40 °C. LAP was added 1 h prior to the (bio)printing steps.

**Table 1:** List of hydrogel groups.

#	Abbreviation	GelMA (%w/v)	Alginate (%w/v)	Ionic crosslinking
				CaCl_2_ (100 mM)
1	GelMA	5	0	−
2	GelMA/Alg 0.1(−)	5	0.1	−
3	GelMA/Alg 0.1(+)	5	0.1	+
4	GelMA/Alg 0.3(−)	5	0.3	−
5	GelMA/Alg 0.3(+)	5	0.3	+
6	GelMA/Alg 0.5(−)	5	0.5	−
7	GelMA/Alg 0.5(+)	5	0.5	+

**Fig. 1:**
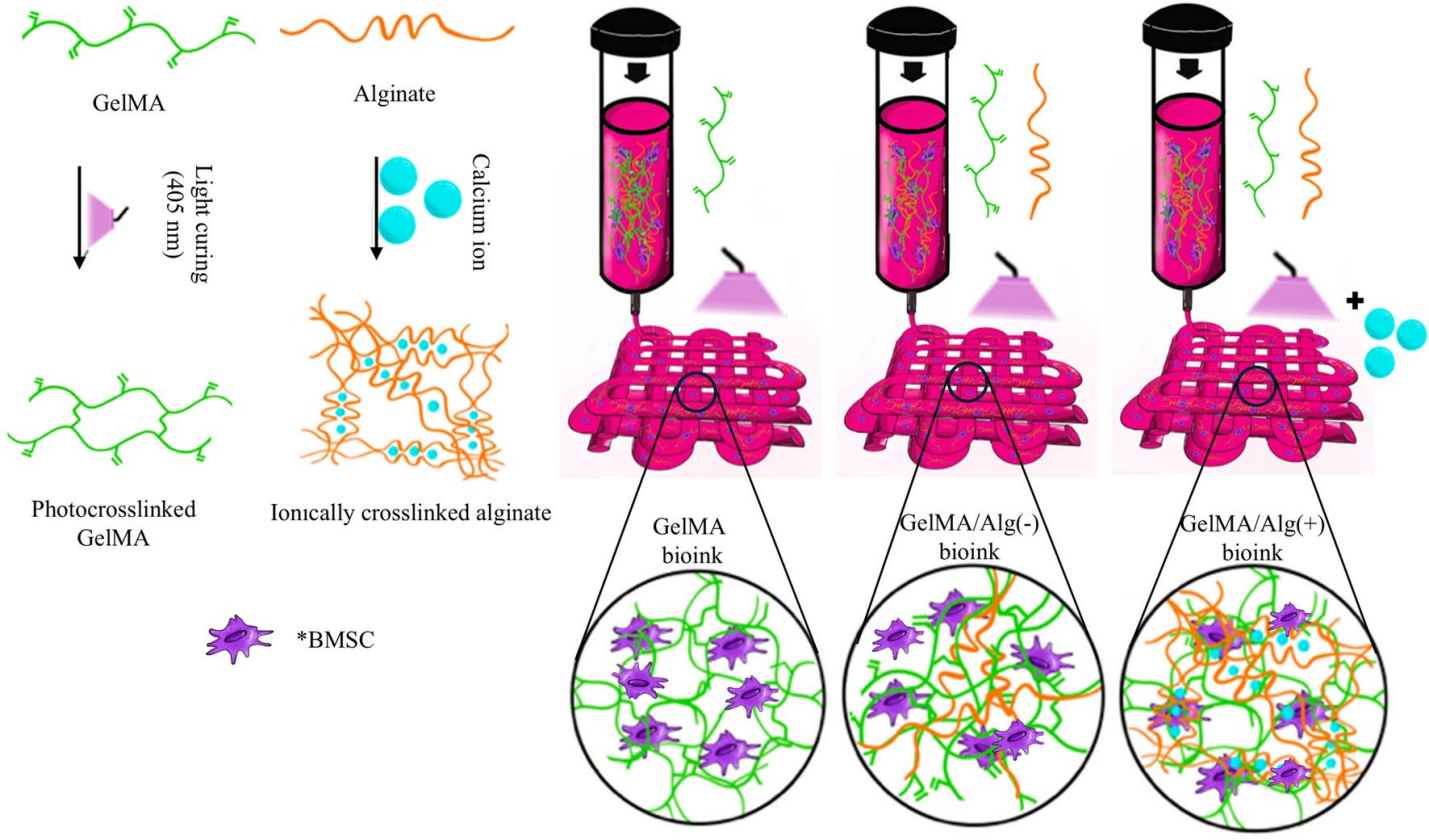
Schematic illustration of the study. In ’’GeMA bioink’’, human bone marrow mesenchymal stem cells (BMSC) are encapsulated in photocrosslinked GelMA. In ’’GeMA/Alg (−) bioink’’, BMSC are encapsulated in photocrosslinked GelMA and non-crosslinked alginate (sacrificial alginate). In ’’GeMA/Alg (+) bioink’’, BMSC are encapsulated in photocrosslinked GelMA and ionically crosslinked alginate.

### Rheological assessment

A rotational rheometer (Discovery Hybrid Rheometer, TA Instruments DHR-2, USA) with a parallel plate geometry with 20 mm steel plates was used to evaluate the rheological properties of the ink. The relationship between the viscosity and temperature of the cell-free inks was investigated at a shear rate of 0.1 s^-1^. The temperature ramp was set from 20 °C to 35 °C, with increments of 1 °C. For further analysis of the viscosity of the inks, the flow sweep test was performed at 26 °C, to evaluate the change in viscosity of each group against shear rate, with a range of 0.1–1000 s^-1^.

The shear thinning behavior of the formulations was further analyzed mathematically by fitting the shear rate-viscosity data with Carreau and Carreau-Yasuda models^37,38^ using following equations:

1 $$Carreau:\mu = \mu_{\infty } + \left( {\mu_{0} - \mu_{\infty } } \right) \left[ {1 + \lambda \dot{\gamma }^{2} } \right]^{{\frac{n - 1}{2}}}$$

2 $${\text{Carreau}} - {\text{Yasuda}}\;\mu = \mu_{\infty } + \left( {\mu_{0} - \mu_{\infty } } \right) \left[ {1 + \lambda \dot{\gamma }^{{\text{a}}} } \right]^{{\frac{{{\text{n}} - 1}}{{\text{a}}}}}$$

where $\dot{\gamma }$ is the shear strain rate, ${\upmu }_{\infty }$ is shear viscosity at an infinite shear rate, ${\upmu }_{0}$ is the shear viscosity at zero shear rate, λ is a time constant representing the fluid’s relaxation time, $n$ is a power law index (often referred to as the "power-law exponent"), and $a$ represents the Yasuda exponent.

### In silico fluidic dynamics analysis

A computational model was used to evaluate the effect of alginate concentration on the shear stress applied by the dispensing nozzle. The geometrical model (stl file) of the needle (0.51 mm inner diameter; EnvisionTEC) was constructed using µCT (Skyscan 1172 x-ray μCT imaging system, Bruker, Kontich, Belgium) at 9 μm resolution, 40 kV and 228 μA). The stl file was then imported to COMSOL Multiphysics 6.0 (COMSOL AB, Sweden) and meshed for computation. The computational model was constructed based on experimental viscosity data obtained from rheological flow ramp tests to improve predictive power. Viscosities of inks were measured across a range of shear rates, and the resulting viscosity-shear rate data were used as inputs for the computational model. These experimentally derived rheological properties were applied to estimate the wall shear stress during the extrusion process. Densities of the formulations were calculated by measuring the mass of 1 mL of the hydrogel precursors. Non-slip boundary conditions were applied. Subsequently, the fluidic dynamics of hydrogel precursors with different alginate concentrations during 3D printing were simulated in COMSOL Multiphysics to predict the pressure, velocity, and fluid shear stress. The edges of the geometries, corresponding to the system input, output, walls, and the initial interface were defined in the boundary conditions. A mesh of 2D triangular type was employed for the simulations, providing an adaptive meshing approach to achieve higher resolution in the extrusion zone of the inks, and effectively reducing the computational load of the simulation.

### Printability and shape fidelity assessment

The prepared inks were transferred into plastic printing cartridges (Nordson FED, USA) and placed first at 4 °C for 20 min. Afterwards, they were placed for 30 min in the Bioplotter (3D-BIOPLOTTER; EnvisionTEC; Gladbeck, Germany) with the temperature set to 26 °C. The platform temperature was adjusted to 10 °C for all printing processes. Boxes (10 mm × 10 mm × 1.28 mm) with 4 layers of perpendicular strands with 1.4 mm in distance, were printed through a straight needle with an inner diameter of 0.51 mm. The stl files were sliced with Prefactory PR software (EnvisionTEC GmbH; Gladbeck, Germany). Printing pressures and speeds were extracted from the fluid dynamic analysis. Two-dimensional top-view images of printed scaffolds were taken using a stereomicroscope (Leica Stereomicroscope M205C, Germany) directly after printing (without crosslinking), and shape fidelity of the inks was evaluated by measuring the spreading ratio (Sr) and printability (Pr) indices of the printed structures. The Sr value shows how much the printed material spreads beyond its intended boundaries during printing and it was measured by dividing the width of printed filament by the inner diameter of the needle^39^. The Pr index was calculated using the following equation^40^:

3 $$\text{Printability}=\frac{{L}^{2}}{16\text{A}}$$

where $\text{L}$ and $\text{A}$ are the perimeter and area of printed interconnected pores, respectively. Under ideal printing, the Sr and Pr indices equal 1.0. ImageJ (Fiji) software (ImageJ 1.53c, NIH, USA) was used to measure the filament width (n = 4), pore areas, and the perimeters of the pores (n = 10).

To assess the buildability and self-standing capacity of the inks, cylinders with 10 mm in height and diameter, and cones with 15 mm in height and 10 mm in diameter were printed.

### Compressive modulus

Bulk cylindrical hydrogels with 8 mm in height and 12 mm in diameter were prepared in cast form for each group. The crosslinking procedure was performed as described in the preparation section. Cylinders were incubated free-floating at 37 °C prior to the compression test for 20 min. A 5944 universal testing machine (Instron, Korea LLC, South Korea) was used to measure the compressive modulus of the hydrogels. The cylinders were compressed to 90% deformation of their thickness at a crosshead speed of 1 mm min^-1^ to obtain a stress-strain curve. The compressive modulus was calculated from the linear region of the stress-strain curve, with a strain range of 0–15%.

### Structural stability and degradation of 3D printed GelMA/Alg

Stability of 3D printed and crosslinked scaffolds was carried out after 14 days of incubation in the cell culture medium based on MEMα-P/S supplemented with 10% FBS (fetal bovine serum; A5256701, Thermo Fisher Scientific, Waltham, Massachusetts, USA) at 37 °C, 5% CO_2_. Following the 14 days, scaffolds were collected for macroscale imaging.

Additionally, 3D printed scaffolds were imaged using stereomicroscope to evaluate deformation of the pores and filaments throughout 14 days of culturing. ImageJ (Fiji) software was used to measure the pore and filament sizes of scaffolds at day 0 and day 14 (n = 5) to calculate the change in the pore area and filament sizes of the scaffolds within 14 days.

To assess the degradation (weight loss), the scaffolds were weighed on days 1, 3, 7, 10, 14, and 21. Day 1 was used to standardize the weight of scaffolds at other time points. The remaining weight over 21 days was calculated according to the following formula:

4 $$\text{Remaining weight }\left({\%}\right)=\left(\frac{{{W}_{1}-W}_{t}}{{W}_{1}}\right)\times 100$$

where ${W}_{1}$ and ${W}_{t}$ are the weights of the scaffolds at day 1 and corresponding time points, respectively.

### Topographical characterization of 3D printed GelMA/Alg

To evaluate the surface characteristics of the various scaffolds investigated in the study, microscale images were taken randomly by scanning electron microscopy implemented with energy dispersive X-ray spectroscopy (SEM–EDX; Leo Supra VP 55, Zeiss, Germany) at day 14 incubation in the growth medium at 37 °C, and 5% CO_2_. Samples were fixed in 3% glutaraldehyde in 0.2 M Na-cacodylate buffer for 30 min, then washed with 0.1 M Na-cacodylate buffer for 20 min and rinsed in distilled water. This was followed by dehydration through a graded series of ethanol from 50%, 70%, 80%, and 100% for 10 min. The samples were mounted on aluminum holders, coated with 70 Å of gold, and examined with SEM–EDX.

### Cell isolation and expansion

Human bone marrow mesenchymal stem cells (BMSC) were used due to their greater relevance to clinical conditions^41,42^. Cell isolation and BMSC characterization were undertaken as previously described^43^. Ethical approval for the isolation of BMSC was granted by the Regional Committee for Medical and Health Research Ethics (REK) in West Norway (REK: 7199/2020, 704,736/2024).

BMSC were expanded in the cell culture medium and incubated in a humidified environment at 37 °C and 5% CO_2_. Trypsinization of cells was carried out using Trypsin–EDTA (ECM0920D; Euroclone, Italy).

### 3D bioprinting

To prepare the bioinks, BMSC with a density of 3 × 10^6^ cells/mL were mixed with the formulations using a luer-lock and syringes. After loading the bioinks into the printing cartridges, they were first incubated for 20 min at 4 °C, and then for 30 min in the Bioplotter with the temperature set to 26 °C. Scaffolds of the same dimension, as presented in the printability section, were bioprinted directly into 6 well-plates. As described in the preparation section, single-crosslinked groups were cured only with visible light of 1200 mW/cm^2^ intensity for two cycles of 30 s. The scaffolds with double-crosslinking were additionally treated with sterile 100 mM CaCl_2_. After 10 min incubation in CaCl_2_, the scaffolds were washed thoroughly with Dulbecco’s phosphate buffered saline (PBS, 14,190–144, Gibco, ThermoFisher Scientific, USA). All crosslinked scaffolds were then transferred into 24 well-plates containing 1 mL culture medium and incubated at 37 °C and 5% CO_2_ for further assays. The medium was changed every two days. All procedures for bioprinting experiments were performed under sterile conditions. The polymer powders were sterilized under UV light for 1 h and the formulations were prepared under a laminar hood. Suspensions of LAP and 100 mM CaCl_2_ solution were sterilized using 0.22 µm filters.

### BMSC responses in 3D bioprinted constructs

LIVE/DEAD™ staining (L3224, Invitrogen, ThermoFisher Scientific) was conducted on the crosslinked scaffolds 3 h after bioprinting to assess the effect of low-concentration alginate and CaCl_2_ treatment on the viability of 3D bioprinted BMSC in different groups. Bioprinted scaffolds were incubated in the staining buffer at 37 °C for 30 min and then washed with PBS. The cells were then imaged using an Andor Dragonfly 5050 high-speed confocal microscope and Fusion software (both from Oxford Instruments, Abingdon, UK). Z-stacks were obtained with steps of 5 μm to a depth of up to 500 μm. Images were acquired with 1024 × 1024 resolution using a high-speed iXon 888 Life EMCCD camera. The viability of the bioprinted cells was then quantitatively analyzed using Image J (Fiji) software by counting the live and dead cells and using the following equation:

5 $$\text{Cell viability }\left({\%}\right)=\frac{\text{Live cells }}{\text{Live cells}+\text{dead cells}}\times 100$$

On day 14 after bioprinting, the scaffolds were fixed with 4% (w/v) paraformaldehyde (PFA, Sigma-Aldrich) for 30 min at room temperature. After permeabilization with 0.1% (v/v) Triton X-100 (Sigma-Aldrich) in PBS for 30 min, samples were introduced to blocking solution consisting of 10% (v/v) normal goat serum (NGS; ab7481, abcam; UK) in PBS for 40 min. Thereafter, a solution of Connexin 43 (Cx43) rabbit monoclonal antibody (1:1000; 83649 T, Cell Signaling Technology®, USA) with 5% (v/v) NGS was added to the scaffolds and incubated overnight at 4 °C. Following that, Cx43 antibody solution was removed, and samples were washed gently with PBS. Goat anti-rabbit secondary antibody (Alexa Flour™ plus 647; A32733, ThermoFisher Scientific) at a concentration of 1:1000 (prepared in PBS) was added to the samples. After overnight incubation in secondary antibody at 4 °C, the samples were stained by Phalloidin Atto 488 (1:500; 49,409; Sigma-Aldrich) for 6 h at room temperature. Eventually, the scaffolds were counterstained with Hoechst 33,342 staining solution (1:2000; Sigma-Aldrich) for 20 min at room temperature. Furthermore, to assess the elongation and migration of BMSC more precisely, 3D bioprinted scaffolds were embedded in Tissue-Tek® O.C.T.TM Compound (Sakura Finetek, Zoeterwonde, Netherlands) overnight at 4^◦^C, followed by consecutive overnight incubation in sucrose 15% (w/v) and 30% (w/v). Subsequently, bioprinted scaffolds were cut using cryotome Cryostat SLEE MNT (SLEE medical GmbH, Nieder-Olm, Germany) into sections 80 µm thick. The sections were stained as described above. 3D scaffolds and sections were imaged using Andor Dragonfly 5050 high-speed confocal microscope and Fusion software. The images were processed using Imaris software (Oxford Instruments, UK).

### Statistical analysis

All statistical analyses were undertaken with GraphPad Prism Software (Version 9.4.1.681, San Diego, USA). The results are shown as mean values ± standard deviation. Multiple comparisons of one-way ANOVA analysis, followed by Tukey’s test, were conducted for all presented data. Statistical significance was defined at the level of p < 0.05.

## Results

### Alginate concentration modulated viscosity and shear thinning variations of inks

**Fig. 2:**
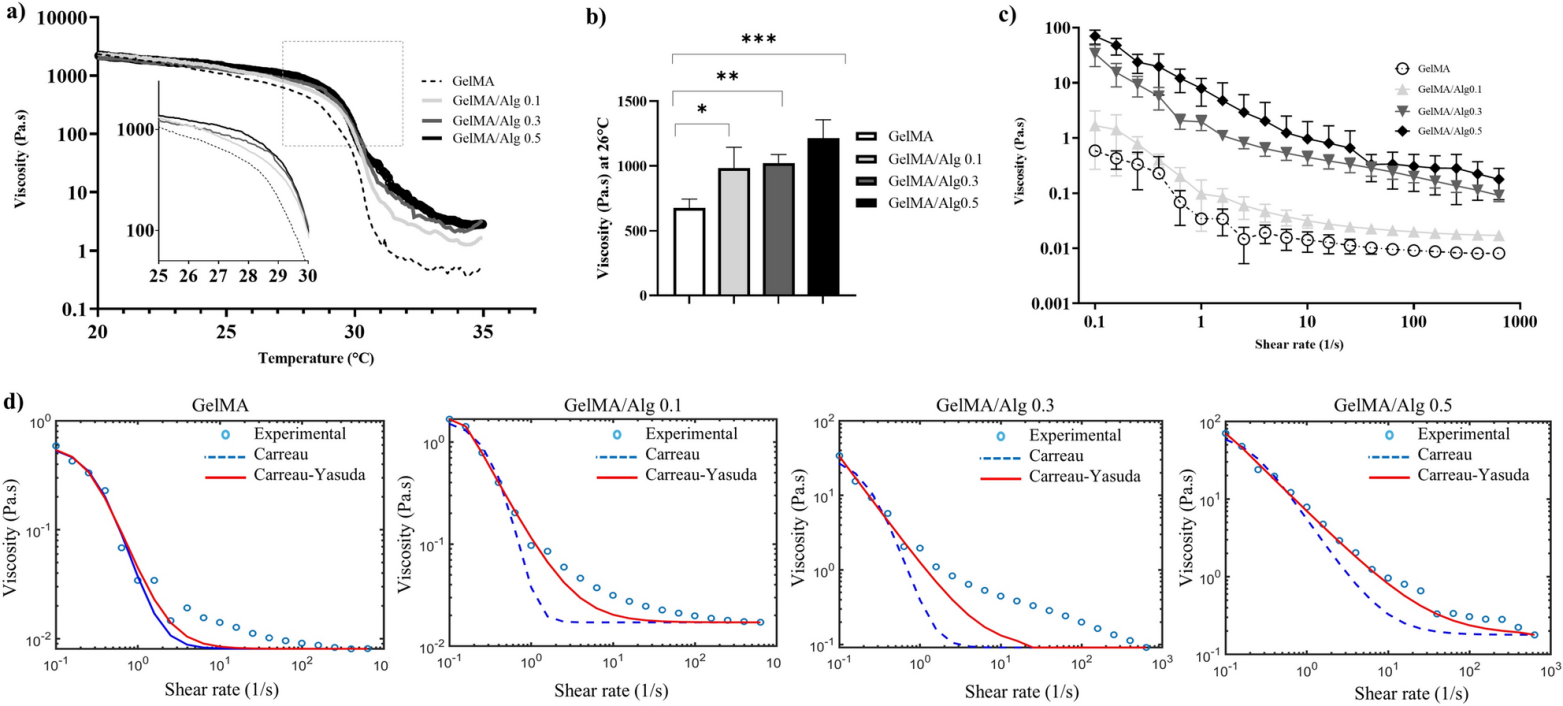
Rheological assessments: (**a**) temperature-dependent viscosity (n = 3). (**b**) viscosity of hydrogel solutions at 26 °C (n = 3), *p ≤ .05, **p ≤ .01, ***p ≤ .001, (**c**) shear-dependent viscosity (n = 3). (**d**) plots fitted to Carreau-Yasuda (solid red line) and Carreau (dash blue line) models compared with experimental values (circles).

The viscosity of all the inks decreased as the temperature increased from 17 to 35 °C (Fig. 2a). Abrupt drops in viscosity were observed at temperatures between 27 and 30 °C. This change in viscosity was more pronounced for the group containing only 5% (w/v) GelMA than in the GelMA/alginate groups. According to results obtained from temperature ramp and minimal temperature fluctuation to the cells, printing temperature throughout the study was set at 26 °C. The average viscosity of formulations at 26 °C indicated that the inclusion of low concentration of alginate in the GelMA matrix led to significant increments in the viscosity of the inks in comparison to GelMA alone (GelMA vs GelMA/Alg 0.1, p = 0.0142; vs GelMA/Alg 0.3, p = 0.0063; vs GelMA/Alg 0.5, p = 0.0003) (Fig. 2b). However, the difference in viscosity between the GelMA/Alg 0.1, GelMA/Alg 0.3, and GelMA/Alg 0.5 groups was not significant (GelMA/Alg 0.1 vs GelMA/Alg 0.5, p = 0.0708).

As an indication of shear thinning behavior, all formulations showed a continuous decrease in the viscosity as shear rate increased (Fig. 2c). Increasing proportion of alginate, even in low concentrations, maximized the shear thinning effect. Viscosity of GelMA/Alg 0.3 and GelMA/Alg 0.5 at 26 °C (printing temperature) and at lower shear rates (0.1- 10 1/s) were substantially higher than the viscosity of GelMA and GelMA/Alg 0.1 (GelMA/Alg 0.3 and GelMA/Alg 0.5 showed approximately 30 and 100 times, respectively, higher viscosity than GelMA). The plots of the Carreau-Yasuda model were fitted to the data of rotational shear viscosity (Fig. 2d). The Carreau-Yasuda model (red line) aligned more closely with the experimental data, especially at lower shear rates. It provided a better fit overall. The Carreau model (blue dashed line) underestimated the viscosity at low shear rates but approached the experimental data more accurately at higher shear rates. Across all formulations, the Carreau-Yasuda model consistently provided higher R^2^ values than Carreau model (Table 2) particularly as alginate concentration increased. Furthermore, a slower response to deformation was observed in Carreau-Yasuda model by predicting longer relaxation times (higher $\lambda$ values) compared to Carreau model, especially for formulations with higher alginate content. The flexibility in capturing more complex rheological behaviors was depicted by varied Yasuda exponent (a) in Carreau-Yasuda model.

**Table 2:** Shear thinning parameters fitted to C-Yasuda and Carreau models (λ: fluid relaxation time, n: power-law exponent, a : Yasuda exponent, R^2^ : fitting index).

Groups	Model	$\lambda (s)$	n	a	R^2^
GelMA	Carreau	2.77	− 1.77	2	0.9884
	C-Yasuda	3.91	− 1.01	2.37	0.9885
GelMA/Alg 0.1	Carreau	2.03	− 4.39	2	0.7796
	C-Yasuda	6.73	− 0.48	9.01	0.8465
GelMA/Alg 0.3	Carreau	3.92	− 2.38	2	0.9436
	C-Yasuda	10.12	− 0.45	139.78	0.9956
GelMA/Alg 0.5	Carreau	5.11	− 0.56	2	0.9683
	C-Yasuda	9.50	− 0.034	86.16	0.9952

### Increasing alginate concentration elevated printing pressure and wall shear stress

**Fig. 3:**
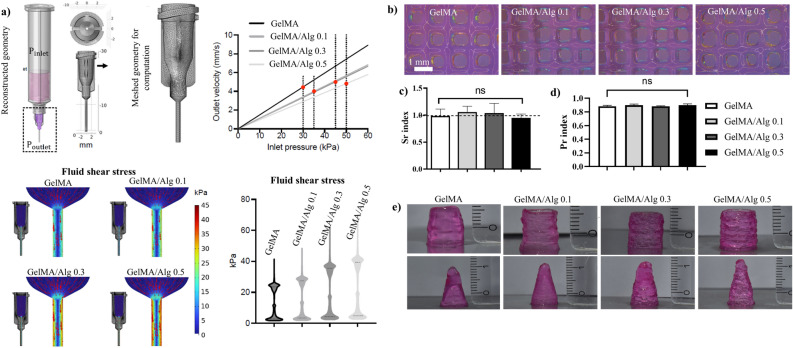
In silico simulation and printability evaluation of the inks: (**a**) in silico simulation of velocity and fluid shear stress applied on the walls of the needle during 3D printing. (**b**) stereomicroscopic images of printed grid boxes (10 mm × 10 mm × 1.28 mm) obtained after printing without crosslinking. (**c**) spreading ratio (Sr) and (**d**) printability (Pr) indices obtained from printed grid boxes (n = 10, ns = not significant). (**e**) cylinders (10 mm height and diameter) and cones (15 mm height and 10 mm diameter) printed with the specified formulations, without crosslinking.

Analysis of in silico fluidic dynamic simulations showed that at a printing speed of 4 mm/sec, the required printing pressure to achieve a consistent flow rate were 30, 35, 45, and 50 kPa for GelMA, GelMA/Alg 0.1, GelMA/Alg 0.3, and GelMA/Alg 0.5, respectively (Fig. 3a). Computed fluid dynamics revealed that, during the extrusion process of the formulations, the shear stress distribution within the nozzle’s geometric structure was higher in the zones adjacent to the nozzle wall (Fig. 3a). Particularly, increasing the concentration of alginate led to a rise in wall shear stress during bioprinting, with estimates of approximately 20 kPa in GelMA alone and over 40 kPa in GelMA/Alg 0.5.

### Enhanced printability and structural integrity of GelMA/alginate inks

The printability and self-standing capacity of the ink formulations were confirmed by printing grid structures with perpendicular filaments and printing large constructs of cylindrical and conical shapes (Fig. 3b–d). Addition of alginate into the GleMA formulation improved the quality of the printed grid structures slightly by minimizing the irregularities and distortion across the grid (Fig. 3b). Printed structures of GelMA/Alg 0.5 appeared well-defined, with clear and consistent outlines for each square within the grid. The uniform thickness and minimal distortion across the grids indicated the good quality of printing.

A minor degree of spreading was observed in GelMA/Alg 0.1 and GelMA/Alg 0.3 by their slight spreading ratio (Sr) index above 1.0 (Fig. 3c). GelMA and GelMA/Alg 0.5 had an Sr of slightly lower than 1.0 but close 1.0, demonstrating a control of spreading. Overall, the spreading ratio across all four inks remained close to 1.0, indicating that none of the inks had excessive spreading. Printability index (Pr) showed that all four formulations had similar affinity to 1, with no statistical difference (ns) in the Pr value (Fig. 3d).

All ink formulations demonstrated sufficient post-printing stability to enable the printing of large structures (cylinders with 10 mm in height and 10 mm in diameter, and cones with 15 mm in height and 10 mm in diameter) without the need for pre- or in situ-crosslinking (Fig. 3e). For the cylindrical shapes, by increasing the concentration of alginate, the surface textures range from relatively smooth to rough with visible layers lines. Formulations of GelMA/Alg 0.3 and GelMA/Alg 0.5 displayed distinct, stacked layers, while GelMA and GelMA/Alg 0.1 appeared more merged. The conical shapes exhibited a similar variation in surface texture, with a slight sloping in GelMA and GelMA/Alg 0.3.

### Ionic crosslinking of alginate enhanced the compression modulus of hydrogels

**Fig. 4:**
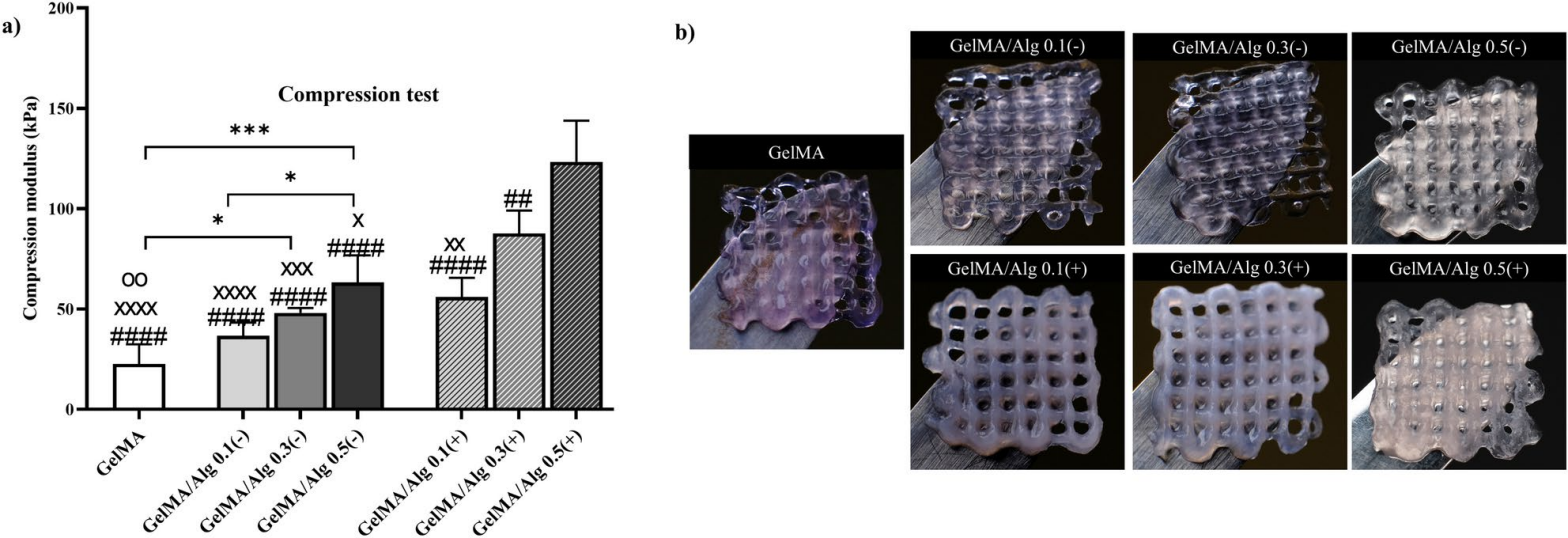
Compression modulus and stability of hydrogels: (**a**) compression modulus of photocrossklinked GelMA and GelMA/Alg bulk cylindrical hydrogels with and without ionic crosslinking of alginate to evaluate the effect of alginate concentration, as well as the effect of ionic-crosslinking; (n = 5), *p ≤ .05, **p ≤ .01, ***p ≤ .001, ****p ≤ .0001; #, × , and o indicate the significant differences of GelMA/Alg 0.5(+), GelMA/Alg 0.3(+), and GelMA/Alg 0.1(+) hydrogel groups, respectively, compared with other groups. (**b**) macroscopic images of 3D printed scaffolds at day 14 of culturing.

There was a general trend where increasing alginate concentration and addition of calcium chloride for alginate crosslinking led to an increase in compression modulus of bulk cylindrical constructs of GelMA/Alg groups (Fig. 4a). Stress-strain plots for each composition are depicted in Supplementary (Figure S1). Although addition of 0.1% (w/v) alginate into GelMA did not change the compression modulus substantially, increasing alginate concentration to 0.3 and 0.5% (w/v) significantly increased the compression modulus from $\sim$ 20 kPa in GelMA to more than 50 kPa in GelMA/Alg 0.3(−) and GelMA/Alg 0.5(−) (GelMA vs. GelMA/Alg 0.3(−), p = 0.0283; vs. GelMA/Alg 0.5(−), p = 0.0001). Furthermore, ionic crosslinking of GelMA/Alg maximized the compressive modulus of calcium-mediated GelMA/Alg constructs significantly compared to their corresponding groups without CaCl_2_ treatment, except in GelMA/Alg 0.1 where ionic crosslinking did not affect the compressive modulus (GelMA/Alg 0.1(−) vs. GelMA/Alg 0.1(+), p = 0.1647; GelMA/Alg 0.3(−) vs. GelMA/Alg 0.3(+), p = 0.0002; GelMA/Alg 0.5(−) vs. GelMA/Alg 0.5(+), p < 0.0001). GelMA/Alg 0.5(+) exhibited the most substantial improvement in compression modulus.

### Slower degradation of ionically crosslinked GelMA/alginate scaffolds

The stability of 3D printed constructs remained overall satisfactory throughout a 14-day culture period irrespective of the experimental groups (Fig. 4b). The GelMA scaffold appeared intact but slightly less defined and softer than the alginate-containing groups. Although GelMA/Alg 0.1(−) had relatively good stability, it showed a weaker structure compared to its double-crosslinked counterpart GelMA/Alg 0.1(+). Both GelMA/Alg0.3(−) and GelMA/Alg0.5(−) demonstrated well-defined structure after 14 days of culturing. Double-crosslinked GelMA/Alg 0.3 and 0.5 had consistent appearance.

**Fig. 5:**
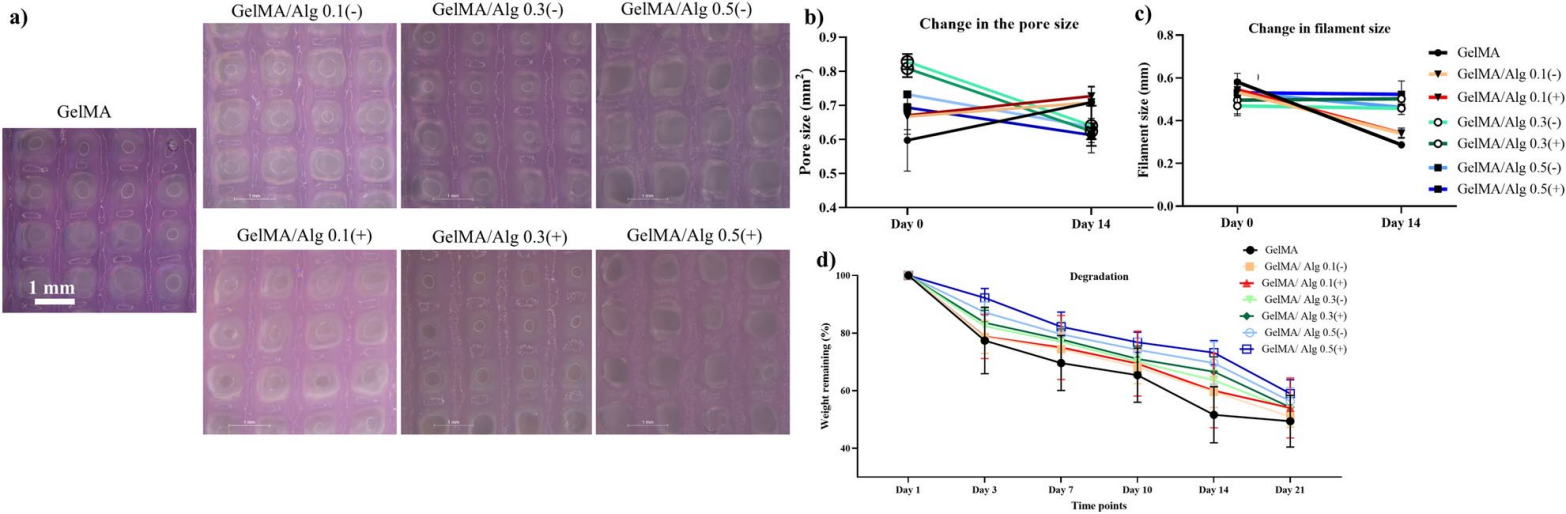
Deformation and degradation of 3D printed scaffolds up to days 14 and 21: (**a**) stereomicroscopic images of 3D printed scaffolds at day 14 of culturing. (**b**) change in the pore area and (**c**) filament size of scaffolds from day 0 to day 14. (**d**) remaining weight (%) of scaffolds up to 21 days (n = 5).

The structural integrity of scaffolds, with different alginate content and for both with/without crosslinking with calcium chloride, remained well-defined throughout 14 days of culturing at 37 °C (Fig. 5a). The pore area of GelMA and GelMA/Alg 0.1 increased by 15% from day 0 to day 14, while GelMA/Alg 0.3 and GelMA/Alg 0.5 groups exhibited 15% decrease in their pore areas (Fig. 5b). On the contrary, the filament size of GelMA and GelMA/Alg 0.1 decreased by 40%, while GelMA/ Alg 0.3 and GelMA/ Alg 0.5 groups had 10% increase in their filament size (Fig. 5c). The weight remaining measurement showed that over 50% of 3D printed GelMA scaffold degraded within 21 days, and this mass loss was substantial compared to GelMA/Alg 0.5 (Fig. 5d). In addition, contribution of ionic crosslinking slightly slowed down the degradation compared to groups without ionic crosslinking. The GelMA/Alg 0.3 group had relatively similar degradation rate compared to the hydrogel composite with 0.5% alginate in both crosslinking conditions, with and without ionic cross linking.

### Increased surface roughness and calcium deposition in ionically crosslinked groups

**Fig. 6:**
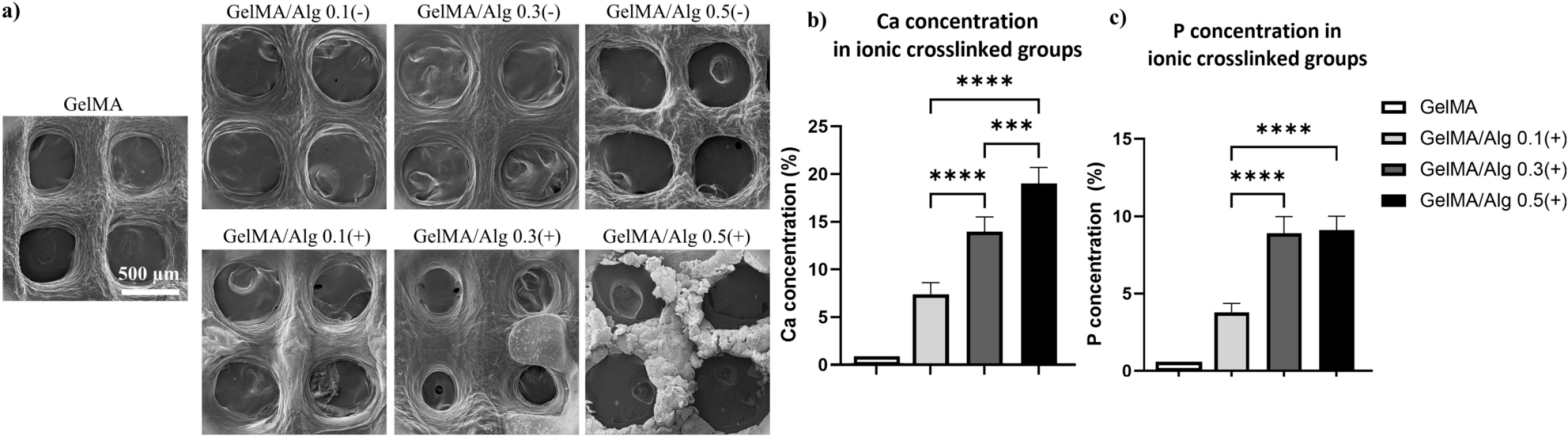
Surface topography of 3D printed scaffolds: (**a**) SEM images 3D printed scaffolds at day 14 of culturing, (**b**,**c**) EDX for quantification of Calcium (Ca) and phosphorous (P) deposition on 3D printed scaffolds (n = 4) *p ≤ .05, **p ≤ .01, ***p ≤ .001, ****p ≤ .0001.

Increasing alginate concentration led to a pronounced rough surface topography across all groups, and ionic crosslinking of alginate resulted in an irregular surface after 14 days of culturing at 37 °C, and 5% CO_2_ (Fig. 6a). The surface of GelMA group appeared relatively smooth, while addition of alginate introduced rougher texture as the alginate concentration increased, particularly in GelMA/Alg 0.5(−). Crosslinking of alginate further introduced rough topography on the surface of the 3D printed scaffolds. A mixed texture of smooth and rough regions was observed in GelMA/Alg 0.3(+) group. The irregularity of the surface morphology was pronounced in calcium-mediated GelMA/Alg 0.5 group with highly heterogeneous and rough regions. On day 14 of culturing, deposition of calcium (Ca) and phosphorous (P) elements were detected in the ionically crosslinked hydrogels (Fig. 6b,c). As the amount of alginate polymers increased, Ca and P deposition was enriched (Ca deposition in GelMA/Alg 0.1(+) vs. GelMA/Alg 0.3(+) and 0.5(+), p < 0.0001; GelMA/Alg 0.3(+) vs. GelMA/Alg 0.5(+), p = 0.0002), (P deposition in GelMA/Alg 0.1(+) vs. GelMA/Alg 0.3(+) and 0.5(+), p = 0.0002 and 0.0001, respectively). The single-crosslinked groups did not show any Ca and P deposition (data not shown).

### Ionic crosslinking of alginate reduced BMSC viability and disrupted cytoskeletal organization

**Fig. 7:**
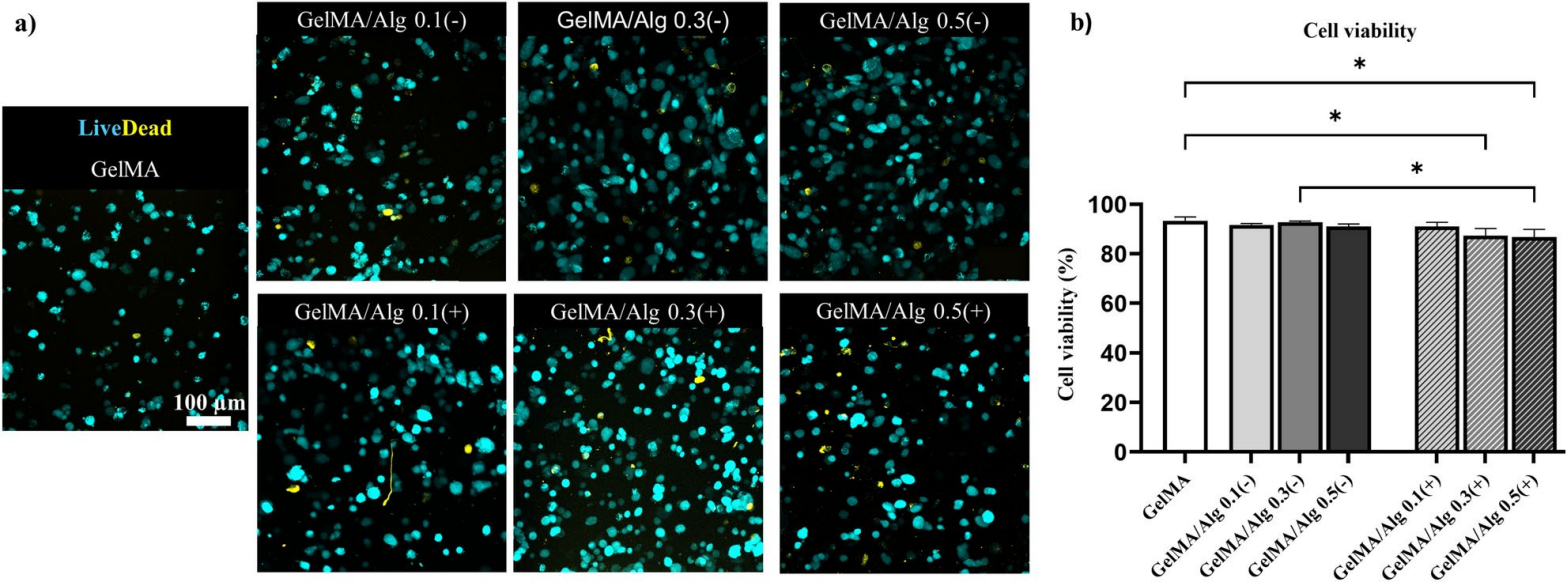
Viability of BMSC: (**a**) LIVE/DEAD staining of 3D bioprinted scaffolds, 3 h after bioprinting (live and dead cells are depicted in cyan and yellow, respectively), (**b**) quantitative evaluation of cell viability obtained from LIVE/DEAD staining (n = 3) *p ≤ .05.

Overall, all experimental groups showed good cell survival 3 h after bioprinting process and culturing at 37 °C, and 5% CO2 (Fig. 7). As the concentration of alginate increased in the GelMA matrix, in both crosslinked and non-crosslinked alginate, there was a slight increase in dead cell presence, although live cells still dominated (Fig. 7a). Addition of alginate concentration did not have a significant impact (ns, p > 0.77) on the viability of bioprinted BMSC and all of them maintained a viability of more than 90% (Fig. 7b). However, ionic crosslinking of the alginate in the GelMA/Alg blend noticeably reduced cell viability down to approximately 85% compared to their non-crosslinked (i.e., photocrosslinked only) counterparts. In particular, GelMA/Alg 0.3(+) and GelMA/Alg 0.5(+) significantly decreased the viability of BMSC compared to GelMA alone (GelMA vs. GelMA/Alg 0.3 (+), p = 0.02; GelMA vs. GelMA/Alg 0.5 (+), p = 0.01) (Fig. 7b). The results indicated that introducing alginate and its ionic crosslinking compromised the optimal environment for cell survival, although the decrease in viability was not drastic.

**Fig. 8:**
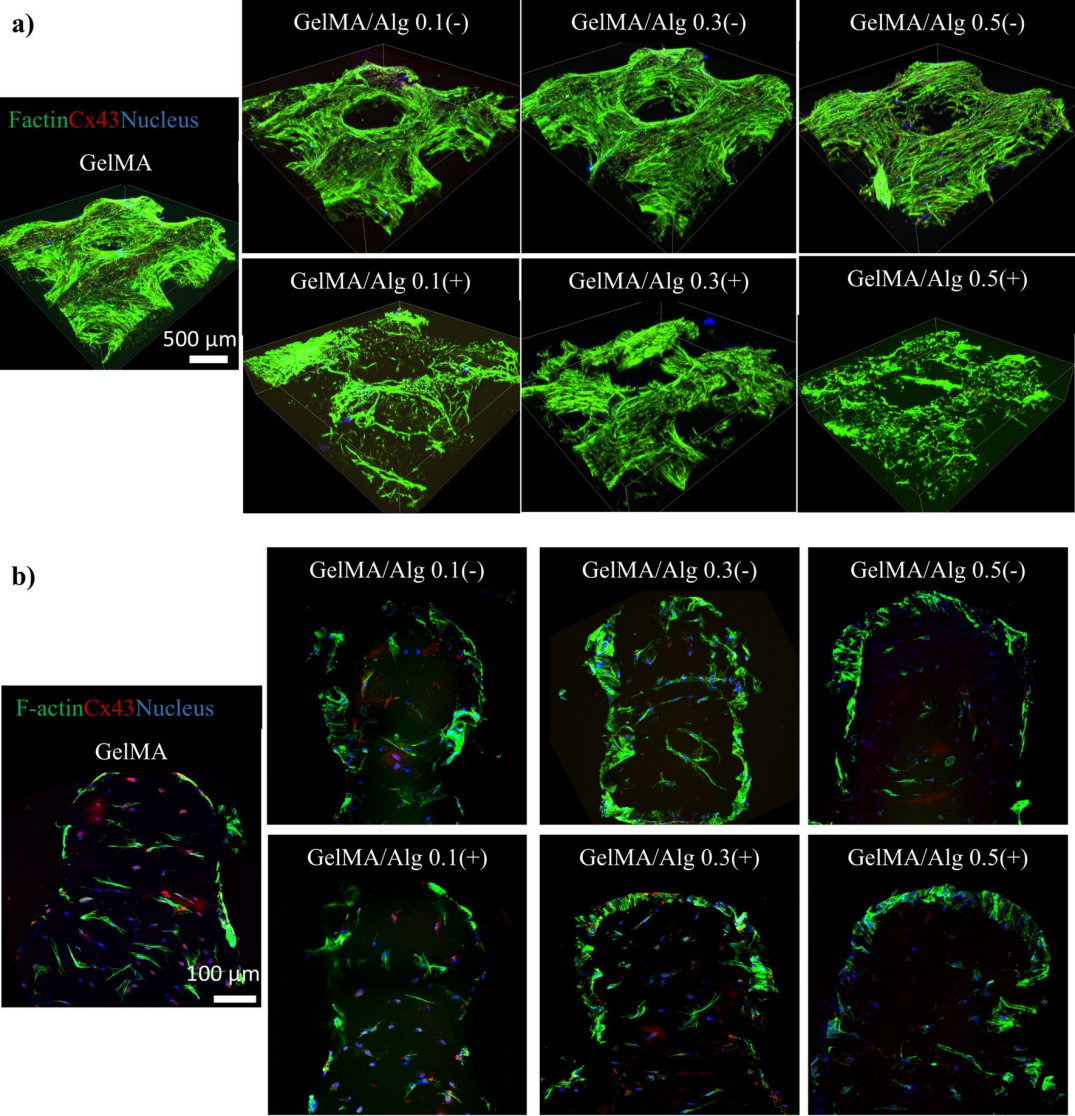
Cytoskeleton morphology of BMSC at day 14 of culturing: (**a**) 3D view of 3D bioprinted BMSC in GelMA and GelMA/Alg scaffolds to evaluate the morphology of BMSC on the surface of the scaffolds. (**b**) Cross-section view of the bioprinted BMSC to evaluate the morphology of BMSC inside the bioprinted constructs (F-actin (green), Cx43 (red), nucleus (blue)).

The 3D views of immunofluorescent staining of bioprinted BMSC showed a dense and interconnected network of filamentous actin (F-actin) in GelMA group indicating robust cytoskeletal organization across the scaffold (Fig. 8). While adding low concentrations of sacrificial alginate (without ionic crosslinking) did not have apparent impact on cell arrangement, there was a decrease in the density and organization of the F-actin network in the calcium-mediated groups (Fig. 8a). The introduction of a low concentration of alginate (0.1 and 0.3) maintained a similar structural organization compared to pure GelMA, with strong F-actin staining that covered the scaffold uniformly. At the highest alginate concentration without ionic crosslinking (GelMA/Alg 0.5(−)), the F-actin network was still present but appeared less dense and slightly dispersed compared to groups with lower alginate concentration. In comparison with the non-crosslinked counterparts, the crosslinked samples showed considerably less continuous and less cohesive F-actin networks, along with disrupted cell organization, suggesting declined cell adhesion and interaction. Particularly, in the group with a higher amount of alginate (GelMA/Alg 0.5), crosslinking of alginate with calcium chloride disrupted the integrity of cell cytoskeleton throughout the scaffolds.

The cross-section view of GelMA showed a relatively even distribution of BMSC with visible F-actin networks and some Cx43 expression inside the hydrogel matrix, indicating moderate cell-cell communication (Fig. 8b). At 0.1 and 0.3% (w/v) alginate without ionic crosslinking (GelMA/Alg 0.1(−) and GelMA/Alg 0.3(−)) BMSC appeared more dispersed and defined compared to GelMA/Alg 0.5(−). Calcium-mediated groups demonstrated disorganized cell alignment with more void spaces appearing inside the matrix compared with non-crosslinked groups.

GelMA/Alg 0.5, both with and without calcium treatment, showed the least distinct cytoskeletal organization, with minimal expression of Cx43, indicating reduced intracellular compared to lower alginate concentration. Furthermore, the results exhibited that lower concentrations of alginate (0.1 and 0.3% w/v) without ionic crosslinking provided a better environment for cell adhesion, cytoskeletal organization, and intracellular communication compared to calcium-mediated groups.

## Discussion

Biofabrication of a suitable microenvironment, in which cells can elongate and proliferate, requires a comprehensive understanding of the properties of bioinks used in the extrusion-based 3D bioprinting technique^44^. GelMA-based bioinks have been extensively evaluated in 3D bioprinting because of their exceptional biocompatibility, thermo- and photoresponsive characteristics and adjustable physicochemical properties, which provide a microenvironment mimicry to the ECM^45–47^. It has been suggested that a low concentration of GelMA provides suitable cell-laden bioinks in terms of cell stability and viability^13^. However, bioprinting of uniformly stacked filaments, using low concentrations of GelMA (< 7% w/v) in pure form is challenging especially when bioprinting temperature is set above 20 °C in order to minimize the impact on cell health^48–51^. In this study, we demonstrated that all low-density formulations, GMP-grade GelMA alone (5% w/v) as well as GelMA blended with alginate (< 0.5% w/v alginate), had successful layer stacking to form 3D constructs, post-printing shape fidelity, processability, and stability at 26 °C. This can be attributed to the high molecular weight of GelMA used in this study (160 kDa), which offers an effective helix formation and as a result, enhancement of the viscosity and stability of GelMA during and after the (bio)printing process^52^. In addition, all four ink formulations in the present study indicated that when the shear rate exceeded the low shear rate threshold (0.1 s^−1^), the resulting shear stress became sufficient to break physical networks within the hydrogel solutions, facilitating smooth extrusion through the printing nozzle. In most studies, GelMA is incorporated with other components (such as alginate, gelatin, methacrylated hyaluronic acid) and apart from photocuring, is additionally crosslinked to compensate for its low viscosity and mechanical strength^13,20,53^. In agreement with previous reports on GelMA/alginate hydrogels, the current study demonstrated higher compressive modulus of samples with higher concentration of alginate; and double-crosslinking (i.e., photocrosslinked and ionically crosslinked) of the hydrogel blends had a higher compressive modulus than solely photocrosslinked hydrogel^32,36,54,55^. The higher compressive modulus of double-crosslinked groups can be associated with the nature of covalent and ionic crosslinking, which occur due to a photo-mediated reaction and chelate effects, respectively. At the microscopic level, the ionic crosslinking tends to force the structural units of alginate to form a compact structure, leading to a maximized rigidity and consequently an enhanced compressive modulus^56^. However, the conformation and rigidity of intra-cluster multimer of ionically crosslinked alginate tend to change in vitro over time by the equilibrium between calcium ions and the monovalent ions in the surrounding solution and eventually causes a fluctuation in the strength of alginate gels^57,58^.

As the core aim of bioprinting is to achieve a proper cell-laden matrix with balanced mechanical properties, structural stability, and biological properties, it is important to evaluate the fate of encapsulated cells. Presence of GelMA in bioprinted constructs was shown to be beneficial as it provides elements that facilitate cell adhesion and cell migration, including integrin-binding motifs and matrix metalloproteinase groups, allowing the encapsulated cells to remodel the surrounding environment to create engineered tissues^59,60^. We demonstrated that the 3D bioprinted BMSC in pure GelMA and in low concentration of sacrificial alginate had higher cell viability and interconnected cellular network compared to the double-crosslinked (photocured and ionic crosslinked) GelMA/alginate bioprinted constructs. Supported by previous studies, bioprinted constructs with higher alginate content had relatively lower cell viability, and less spreading and cell-cell connection inside the scaffolds, which may be attributed to the reversible inhibition effect of alginate on cells arising from its lack of cell adhesion motifs, high rigidity, limited degradability, restricted nutrient diffusion, and disrupted calcium ion interference^22,40,61^.

When alginate is included in the composition, the concentration of CaCl_2_ and the crosslinking duration of cell-laden alginate should be carefully considered in order to achieve optimal 3D bioprinted constructs with minimal compromise of cell viability. Treating alginate-containing bioprinted constructs with CaCl_2_ solution for a longer time (30 min) had a negative impact on cell survival^31^. The damage and cell loss were associated with the osmotic effect caused by ionic solution^62^. Moreover, high calcium concentration can disturb the state of cell electrolytes and lead to cell membrane damage^63^. Calcium imbalance in cells can severely impact cellular mitochondria^64^. When mitochondria, which typically maintain low calcium levels, absorbs excess calcium, it triggers a cascade of harmful effects, including mitochondrial membrane swelling, disruption of normal respiratory processes, and a substantial reduction in the adenosine triphosphate (ATP) level. The influx of calcium ions can disrupt the balance of forces that normally maintain cell shape, leading to the formation of membrane protrusions known as blebs. This bleb formation is often associated with the changes in the actin cytoskeleton^65^. Similarly, we observed a severe change in the cell orientation of the calcium-mediated groups, particularly in the group with higher amount of alginate (GeMA/Alg 0.5), which can be attributed to this bleb formation.

Furthermore, alginate-containing hydrogels which are ionically crosslinked may lose their stability in the physiological condition because the calcium ions of the crosslinking reagent may be removed in the presence of calcium chelators (e.g. phosphates), monovalent ions (e.g. K^+^, Na^+^) and non-crosslinking divalent ions (Mg^2+^)^19^. In this study, we showed that treating alginate-containing 3D bioprinted constructs with CaCl_2_ affected the surface topography of the scaffolds. The deposition of calcium and phosphorus elements can result in rough, irregular, and heterogeneous surface features^66^. If deposition becomes uncontrolled and excessive, it can disrupt the cell cytoskeleton integrity by impairing the focal adhesion dynamics, leading to uneven cell spreading^67–70^. This aligns with our findings in calcium-mediated groups, where the uncontrolled Ca-P deposition (9%, 15%, and 20% calcium deposition in GelMA/Alg 0.1(+), GelMA/Alg 0.3(+), and 0.5(+), respectively. 4%, 9%, and 9% phosphorus deposition in GelMA/Alg 0.1(+)) caused disruption in cell arrangements and cell cytoskeletal structure. Moreover, double-crosslinking of GelMA/alginate blends with photocuring and CaCl_2_ resulted in a stiffer surface which inhibits the penetration of nutrients and oxygen into the bioprinted scaffolds^71,72^.

We found that over 21 days, the stability and mass loss of groups with sacrificial alginate were comparable to those of Ca^2+^-treated groups. This phenomenon may be attributed to the entrapment of the alginate chains inside the GelMA matrix due to the formation of interpenetrating networks, which has been shown to be effective in enhancing the compressive stiffness and toughness of the entangled polymers^36,73,74^. Another factor influencing the comparable stability of single and double-crosslinked groups may be the low concentration of calcium ions available in the culture medium (~ 1.8 mM)^63^, which can gradually and harmlessly crosslink alginate-containing polymers. Therefore, by utilizing the interpenetrating network formation of GelMA/alginate and the low concentration of calcium ions supplementing the culture medium, the additional and external ionic crosslinking of alginate in the bioprinting process of GelMA/alginate hydrogel composites can be eliminated. As a result, the potential damage caused by excessive calcium ions used for ionic crosslinking of alginate can be minimized. Alternatively, covalent crosslinking methods can be used to minimize the adverse effects of ionic crosslinking of alginate^75–77^. For instance, ’’click’’ reactions have emerged as an effective method for creating alginate-based hydrogels with minimum harmful side effects^75^. Vu et al. designed injectable, reduction-responsive hydrogels using norbornene-substituted alginate and a water-soluble disulfide crosslinker through inverse electron demand Diels–Alder (IEDDA) reaction between norbornene (Nb) and tetrazine groups (Tz), and their cell culture experiments confirmed the non-toxicity of both crosslinker and hydrogels to fibroblast cells. In addition, it was shown that the bioprinting of fibroblasts in photocrosslinkable microgel alginate, an alginate derivative containing tyramine moiety^54^, had high proliferation and migration abilities of living cells^76,77^.

While our in vitro tests provided valuable insights, the mechanical stability, retention, and degradation patterns observed may not fully reflect in vivo conditions, necessitating further investigation in clinical systems. Additionally, the small scale and simple geometry of our experimental models may not accurately represent clinical scenarios, underscoring the need for more extensive research using larger and more complex structures that better simulate clinical applications.

## Conclusion

In this study, BMSC were 3D bioprinted in GelMA-based bioinks, using extrusion-based bioprinting. All formulations of GelMA alone and GelMA/alginate blends, with low concentrations of GelMA (5% w/v) and alginate (i.e., 0.1, 0.3, and 0.5% w/v), showed appropriate shear thinning behavior as a bioink, and the printability and fidelity of these groups were comparable. Large-dimensional constructs were successfully printed with these low-density formulations, demonstrating their self-standing capacity. In addition, the 3D bioprinted scaffolds without ionic crosslinking had degradation rates relatively similar to those of double-crosslinked (photocrosslinked and ionically crosslinked) printed scaffolds. Both the inclusion of low concentration of alginate (0.3, 0.5% w/v) into the GelMA matrix and double-crosslinking of the materials improved the mechanical properties of the hydrogels. The ionic crosslinking of the bioprinted constructs changed the scaffolds’ surface morphology by deposition of calcium and phosphorous, which affected cell spreading throughout the scaffolds. These results suggest that the inclusion of low-concentration alginate (0.3% w/v), without external ionic crosslinking of alginate, is sufficient to provide balanced structural support and cell interaction. With this approach, the extra post-printing step for ionic crosslinking of alginate can be eliminated to minimize cell damage and simplify the bioprinting process for clinical applications.

## Supplementary Information

Supplementary Information.

## Data Availability

All raw data supporting the findings of this study will be made available upon request to the corresponding author without reservation.

## References

[1] Blaeser, A. et al. Controlling shear stress in 3D Bioprinting is a key factor to balance printing resolution and stem cell integrity. *Adv. Healthc. Mater.* **5**, 326–333 (2016). 26626828 10.1002/adhm.201500677

[2] Groll, J. et al. A definition of bioinks and their distinction from biomaterial inks. *Biofabrication* **11**, 013001 (2019). 10.1088/1758-5090/aaec52 30468151

[3] Murphy, S. V. & Atala, A. 3D bioprinting of tissues and organs. *Nat. Biotechnol.* **32**, 773–785 (2014). 25093879 10.1038/nbt.2958

[4] Moroni, L. et al. Biofabrication strategies for 3D in vitro models and regenerative medicine. *Nat. Rev. Mater.* **3**, 21–37 (2018). 31223488 10.1038/s41578-018-0006-y PMC6586020

[5] Osidak, E. O., Kozhukhov, V. I., Osidak, M. S. & Domogatsky, S. P. Collagen as bioink for bioprinting: A comprehensive review. *Int. J. Bioprint.* **6**, 1–10 (2020). 10.18063/ijb.v6i3.270 PMC7557346 33088985

[6] Ahmadi Soufivand, A., Faber, J., Hinrichsen, J. & Budday, S. Multilayer 3D bioprinting and complex mechanical properties of alginate-gelatin mesostructures. *Sci. Rep.* **13**, 1–14 (2023). 37438423 10.1038/s41598-023-38323-2 PMC10338535

[7] Kang, D. et al. 3D bioprinting of a gelatin-alginate hydrogel for tissue-engineered hair follicle regeneration. *Acta Biomater.* **165**, 19–30 (2023). 35288311 10.1016/j.actbio.2022.03.011

[8] Das, S. et al. Bioprintable, cell-laden silk fibroin-gelatin hydrogel supporting multilineage differentiation of stem cells for fabrication of three-dimensional tissue constructs. *Acta Biomater.* **11**, 233–246 (2015). 25242654 10.1016/j.actbio.2014.09.023

[9] Derakhshanfar, S. et al. 3D bioprinting for biomedical devices and tissue engineering: A review of recent trends and advances. *Bioact. Mater.* **3**, 144–156 (2018). 29744452 10.1016/j.bioactmat.2017.11.008 PMC5935777

[10] Williams, D., Thayer, P., Martinez, H., Gatenholm, E. & Khademhosseini, A. A perspective on the physical, mechanical and biological specifications of bioinks and the development of functional tissues in 3D bioprinting. *Bioprinting* **9**, 19–36 (2018).

[11] Gungor-Ozkerim, P. S., Inci, I., Zhang, Y. S., Khademhosseini, A. & Dokmeci, M. R. Bioinks for 3D bioprinting: An overview. *Biomater. Sci.* **6**, 915–946 (2018). 29492503 10.1039/c7bm00765e PMC6439477

[12] Gillispie, G. et al. Assessment methodologies for extrusion-based bioink printability. *Biofabrication* **12**, 022003 (2020). 31972558 10.1088/1758-5090/ab6f0d PMC7039534

[13] Yin, J., Yan, M., Wang, Y., Fu, J. & Suo, H. 3D Bioprinting of Low-Concentration Cell-Laden Gelatin Methacrylate (GelMA) Bioinks with a Two-Step Cross-linking Strategy. *ACS Appl. Mater. Interfaces* **10**, 6849–6857 (2018). 29405059 10.1021/acsami.7b16059

[14] Luo, Y., Chen, B., Zhang, X., Huang, S. & Wa, Q. 3D printed concentrated alginate/GelMA hollow-fibers-packed scaffolds with nano apatite coatings for bone tissue engineering. *Int. J. Biol. Macromol.* **202**, 366–374 (2022). 35063479 10.1016/j.ijbiomac.2022.01.096

[15] Shirahama, H., Lee, B. H., Tan, L. P. & Cho, N. J. Precise tuning of facile one-pot gelatin methacryloyl (GelMA) synthesis. *Sci. Rep.* **6**, 1–11 (2016). 27503340 10.1038/srep31036 PMC4977492

[16] Moon, S. H. et al. Photocrosslinkable natural polymers in tissue engineering. *Front. Bioeng. Biotechnol.* **11**, 1–18 (2023). 10.3389/fbioe.2023.1127757 PMC10037533 36970625

[17] Yue, K. et al. Synthesis, properties, and biomedical applications of gelatin methacryloyl (GelMA) hydrogels. *Biomaterials* **73**, 254–271 (2015). 26414409 10.1016/j.biomaterials.2015.08.045 PMC4610009

[18] Loessner, D. et al. Functionalization, preparation and use of cell-laden gelatin methacryloyl–based hydrogels as modular tissue culture platforms. *Nat. Protoc.* **11**, 727–746 (2016). 26985572 10.1038/nprot.2016.037

[19] Kuo, C. K. & Ma, P. X. Maintaining dimensions and mechanical properties of ionically crosslinked alginate hydrogel scaffolds in vitro. *J. Biomed. Mater. Res. Part A* **84**, 899–907 (2008). 10.1002/jbm.a.31375 17647237

[20] Aldana, A. A., Valente, F., Dilley, R. & Doyle, B. Development of 3D bioprinted GelMA-alginate hydrogels with tunable mechanical properties. *Bioprinting* **21**, e00105 (2021).

[21] Seyedmahmoud, R. et al. Three-dimensional bioprinting of functional skeletal muscle tissue using gelatin. *Micromachines* **10**, 1–12 (2019). 10.3390/mi10100679 PMC6843821 31601016

[22] Zhang, J. et al. Alginate dependent changes of physical properties in 3D bioprinted cell-laden porous scaffolds affect cell viability and cell morphology. *Biomed. Mater.* **14**, 065009 (2019). 31426033 10.1088/1748-605X/ab3c74

[23] Malda, J. et al. 25th anniversary article: Engineering hydrogels for biofabrication. *Adv. Mater.* **25**, 5011–5028 (2013). 24038336 10.1002/adma.201302042

[24] Rashad, A., Mustafa, K., Heggset, E. B. & Syverud, K. Cytocompatibility of Wood-derived cellulose nanofibril hydrogels with different surface chemistry. *Biomacromolecules* **18**, 1238–1248 (2017). 28263573 10.1021/acs.biomac.6b01911

[25] Nützl, M., Schrottenbaum, M., Müller, T. & Müller, R. Mechanical properties and chemical stability of alginate-based anisotropic capillary hydrogels. *J. Mech. Behav. Biomed. Mater.* **134**, 105397 (2022). 35932645 10.1016/j.jmbbm.2022.105397

[26] Schütz, K. et al. Three-dimensional plotting of a cell-laden alginate/methylcellulose blend: towards biofabrication of tissue engineering constructs with clinically relevant dimensions. *J. Tissue Eng. Regen. Med.* **11**, 1574–1587 (2017). 26202781 10.1002/term.2058

[27] Lee, K. Y. & Mooney, D. J. Alginate: Properties and biomedical applications. *Prog. Polym. Sci.* **37**, 106–126 (2012). 22125349 10.1016/j.progpolymsci.2011.06.003 PMC3223967

[28] Gillette, B. M., Jensen, J. A., Wang, M., Tchao, J. & Sia, S. K. Dynamic hydrogels: Switching of 3D microenvironments using two-component naturally derived extracellular matrices. *Adv. Mater.* **22**, 686–691 (2010). 20217770 10.1002/adma.200902265

[29] Orrenius, S., McCabe, M. J. J. & Nicotera, P. Ca(2+)-dependent mechanisms of cytotoxicity and programmed cell death. *Toxicol. Lett.* **64–65**, 357–364 (1992). 10.1016/0378-4274(92)90208-2 1335178

[30] Demaurex, N. & Distelhorst, C. Apoptosis–the calcium connection. *Science* **300**, 65–67 (2003). 12677047 10.1126/science.1083628

[31] Cao, N., Chen, X. B. & Schreyer, D. J. Influence of calcium ions on cell survival and proliferation in the context of an alginate hydrogel. *ISRN Chem. Eng.* **2012**, 1–9 (2012).

[32] Marfoglia, A., Tibourtine, F., Pilloux, L. & Cazalbou, S. Tunable double-network GelMA/alginate hydrogels for platelet lysate-derived protein delivery. *Bioengineering* **10**, 1044 (2023). 37760147 10.3390/bioengineering10091044 PMC10525654

[33] Li, J. et al. Development and systematic characterization of GelMA/alginate/PEGDMA/xanthan gum hydrogel bioink system for extrusion bioprinting. *Biomaterials* **293**, 121969 (2023). 36566553 10.1016/j.biomaterials.2022.121969 PMC9868087

[34] Shams, E., Barzad, M. S., Mohamadnia, S., Tavakoli, O. & Mehrdadfar, A. A review on alginate-based bioinks, combination with other natural biomaterials and characteristics. *J. Biomater. Appl.* **37**, 355–372 (2022). 35510845 10.1177/08853282221085690

[35] Ahmad Raus, R., Wan Nawawi, W. M. F. & Nasaruddin, R. R. Alginate and alginate composites for biomedical applications. *Asian J. Pharm. Sci.* **16**, 280–306 (2021). 34276819 10.1016/j.ajps.2020.10.001 PMC8261255

[36] Wang, B. et al. Affinity-bound growth factor within sulfated interpenetrating network bioinks for bioprinting cartilaginous tissues. *Acta Biomater.* **128**, 130–142 (2021). 33866035 10.1016/j.actbio.2021.04.016

[37] Byron Bird, R. & Carreau, P. J. A nonlinear viscoelastic model for polymer solutions and melts-I. *Chem. Eng. Sci.* **23**, 427–434 (1968).

[38] Yasuda, K. Investigation of the analogies between viscometric and linear viscoelastic properties of polystyrene fluids (1979).

[39] Ojansivu, M. et al. Wood-based nanocellulose and bioactive glass modified gelatin-alginate bioinks for 3D bioprinting of bone cells. *Biofabrication* **11**, 035010 (2019). 30754034 10.1088/1758-5090/ab0692

[40] Ouyang, L., Yao, R., Zhao, Y. & Sun, W. Effect of bioink properties on printability and cell viability for 3D bioplotting of embryonic stem cells. *Biofabrication* **8**, 035020 (2016). 27634915 10.1088/1758-5090/8/3/035020

[41] Ong, C. S. et al. 3D bioprinting using stem cells. *Pediatr. Res.* **83**, 223–231 (2018). 28985202 10.1038/pr.2017.252

[42] Tasoglu, S. & Demirci, U. Bioprinting for stem cell research. *Trends Biotechnol.* **31**, 10–19 (2013). 23260439 10.1016/j.tibtech.2012.10.005 PMC3534918

[43] Mohamed-Ahmed, S. et al. Adipose-derived and bone marrow mesenchymal stem cells: a donor-matched comparison. *Stem Cell Res. Ther.* **9**, 168 (2018). 29921311 10.1186/s13287-018-0914-1 PMC6008936

[44] Ozbolat, I. T. & Hospodiuk, M. Current advances and future perspectives in extrusion-based bioprinting. *Biomaterials* **76**, 321–343 (2016). 26561931 10.1016/j.biomaterials.2015.10.076

[45] Ying, G., Jiang, N., Yu, C. & Zhang, Y. S. Three-dimensional bioprinting of gelatin methacryloyl (GelMA). *Bio-Des. Manuf.* **1**, 215–224 (2018).

[46] Jain, T. et al. Impact of cell density on the bioprinting of gelatin methacrylate (GelMA) bioinks. *Bioprinting* **22**, e00131 (2021).

[47] Liu, C. et al. 3D bioprinting of cell-laden nano-attapulgite/gelatin methacrylate composite hydrogel scaffolds for bone tissue repair. *J. Mater. Sci. Technol.* **135**, 111–125 (2023).

[48] Schuurman, W. et al. Gelatin-methacrylamide hydrogels as potential biomaterials for fabrication of tissue-engineered cartilage constructs. *Macromol. Biosci.* **13**, 551–561 (2013). 23420700 10.1002/mabi.201200471

[49] Billiet, T., Gevaert, E., De Schryver, T., Cornelissen, M. & Dubruel, P. The 3D printing of gelatin methacrylamide cell-laden tissue-engineered constructs with high cell viability. *Biomaterials* **35**, 49–62 (2014). 24112804 10.1016/j.biomaterials.2013.09.078

[50] Celikkin, N. et al. In vitro and in vivo assessment of a 3D printable gelatin methacrylate hydrogel for bone regeneration applications. *J. Biomed. Mater. Res. Part B* **110**, 2133–2145 (2022). 10.1002/jbm.b.35067 35388573

[51] Bertassoni, L. E. et al. Direct-write bioprinting of cell-laden methacrylated gelatin hydrogels. *Biofabrication* **6**, 024105 (2014). 24695367 10.1088/1758-5082/6/2/024105 PMC4040163

[52] Elharfaoui, N., Djabourov, M. & Babel, W. Molecular weight influence on gelatin gels: Structure, enthalpy and rheology. *Macromol. Symp.* **256**, 149–157 (2007).

[53] Schweinitzer, S., Kadousaraei, M. J., Aydin, M. S., Mustafa, K. & Rashad, A. Measuring cell proliferation in bioprinting research. *Biomed. Mater.* **19**, 031001 (2024). 10.1088/1748-605X/ad3700 38518363

[54] Kim, E., Kim, M. H., Song, J. H., Kang, C. & Park, W. H. Dual crosslinked alginate hydrogels by riboflavin as photoinitiator. *Int. J. Biol. Macromol.* **154**, 989–998 (2020). 32194119 10.1016/j.ijbiomac.2020.03.134

[55] Ansari, S. et al. Regulation of the fate of dental-derived mesenchymal stem cells using engineered alginate-GelMA hydrogels. *J. Biomed. Mater. Res. Part A* **105**, 2957–2967 (2017). 10.1002/jbm.a.36148 PMC5623163 28639378

[56] Chaudhuri, O. et al. Substrate stress relaxation regulates cell spreading. *Nat. Commun.* **6**, 1–7 (2015). 10.1038/ncomms7365 PMC4518451 25695512

[57] Pössl, A., Hartzke, D., Schmidts, T. M., Runkel, F. E. & Schlupp, P. A targeted rheological bioink development guideline and its systematic correlation with printing behavior. *Biofabrication* **13**, 035021 (2021). 10.1088/1758-5090/abde1e 33472177

[58] Fang, Y. et al. Multiple steps and critical behaviors of the binding of calcium to alginate. *J. Phys. Chem. B* **111**, 2456–2462 (2007). 17305390 10.1021/jp0689870

[59] Nichol, J. W. et al. Cell-laden microengineered gelatin methacrylate hydrogels. *Biomaterials* **31**, 5536–5544 (2010). 20417964 10.1016/j.biomaterials.2010.03.064 PMC2878615

[60] Colosi, C. et al. Microfluidic bioprinting of heterogeneous 3D tissue constructs using low-viscosity bioink. *Adv. Mater.* **28**, 677–684a (2016). 26606883 10.1002/adma.201503310 PMC4804470

[61] Hunt, N. C., Shelton, R. M. & Grover, L. M. Reversible mitotic and metabolic inhibition following the encapsulation of fibroblasts in alginate hydrogels. *Biomaterials* **30**, 6435–6443 (2009). 19709739 10.1016/j.biomaterials.2009.08.014

[62] Carvalho, A. F., Gasperini, L., Ribeiro, R. S., Marques, A. P. & Reis, R. L. Control of osmotic pressure to improve cell viability in cell-laden tissue engineering constructs. *J. Tissue Eng. Regen. Med.* **12**, e1063–e1067 (2018). 28342296 10.1002/term.2432

[63] Dong, Z., Saikumar, P., Weinberg, J. M. & Venkatachalam, M. A. Calcium in cell injury and death. *Annu. Rev. Pathol.* **1**, 405–434 (2006). 18039121 10.1146/annurev.pathol.1.110304.100218

[64] Orrenius, S., McConkey, D. J., Bellomo, G. & Nicotera, P. Role of Ca2+ in toxic cell killing. *Trends Pharmacol. Sci.* **10**, 281–285 (1989). 2672472 10.1016/0165-6147(89)90029-1

[65] Nicotera, P., Hartzell, P., Davis, G. & Orrenius, S. The formation of plasma membrane blebs in hepatocytes exposed to agents that increase cytosolic Ca2+ is mediated by the activation of a non-lysosomal proteolytic system. *FEBS Lett.* **209**, 139–144 (1986). 3100326 10.1016/0014-5793(86)81099-7

[66] Costa, D. O. et al. Control of surface topography in biomimetic calcium phosphate coatings. *Langmuir* **28**, 3871–3880 (2012). 22242934 10.1021/la203224a

[67] Mandal, B. B., Das, S., Choudhury, K. & Kundu, S. C. Implication of silk film rgd availability and surface roughness on cytoskeletal organization and proliferation of primary rat bone marrow cells. *Tissue Eng. Part A* **16**, 2391–2403 (2010). 20214452 10.1089/ten.TEA.2009.0206

[68] Anselme, K. & Bigerelle, M. Role of materials surface topography on mammalian cell response. *Int. Mater. Rev.* **56**, 243–266 (2011).

[69] Chang, C. H., Lee, H. H. & Lee, C. H. Substrate properties modulate cell membrane roughness by way of actin filaments. *Sci. Rep.* **7**, 1–11 (2017). 28831175 10.1038/s41598-017-09618-y PMC5567215

[70] Anselme, K. et al. The relative influence of the topography and chemistry of TiAl6V4 surfaces on osteoblastic cell behaviour. *Biomaterials* **21**, 1567–1577 (2000). 10885729 10.1016/s0142-9612(00)00042-9

[71] Jang, J. et al. Effects of alginate hydrogel cross-linking density on mechanical and biological behaviors for tissue engineering. *J. Mech. Behav. Biomed. Mater.* **37**, 69–77 (2014). 24880568 10.1016/j.jmbbm.2014.05.004

[72] Sonaye, S. Y., Ertugral, E. G., Kothapalli, C. R. & Sikder, P. Extrusion 3D (Bio)printing of alginate-gelatin-based composite scaffolds for skeletal muscle tissue engineering. *Materials* **15**, 7945 (2022). 36431432 10.3390/ma15227945 PMC9695625

[73] Hong, S. et al. 3D printing of highly stretchable and tough hydrogels into complex, cellularized structures. *Adv. Mater.* **27**, 4035–4040 (2015). 26033288 10.1002/adma.201501099 PMC4849481

[74] Liao, I. C., Moutos, F. T., Estes, B. T., Zhao, X. & Guilak, F. Composite three-dimensional woven scaffolds with interpenetrating network hydrogels to create functional synthetic articular cartilage. *Adv. Funct. Mater.* **23**, 5833–5839 (2013). 24578679 10.1002/adfm.201300483 PMC3933181

[75] Anugrah, D. S. B., Ramesh, K., Kim, M., Hyun, K. & Lim, K. T. Near-infrared light-responsive alginate hydrogels based on diselenide-containing cross-linkage for on demand degradation and drug release. *Carbohydr. Polym.* **223**, 115070 (2019). 31427031 10.1016/j.carbpol.2019.115070

[76] Lee, S. et al. Visible light-crosslinkable tyramine-conjugated alginate-based microgel bioink for multiple cell-laden 3D artificial organ. *Carbohydr. Polym.* **313**, 120895 (2023). 37182936 10.1016/j.carbpol.2023.120895

[77] Kim, E. et al. Silk fibroin enhances cytocompatibilty and dimensional stability of alginate hydrogels for light-based three-dimensional bioprinting. *Biomacromolecules* **22**, 1921–1931 (2021). 33840195 10.1021/acs.biomac.1c00034

