# Supplementary material for: Bioprinting of mesenchymal stem cells in low concentration gelatin methacryloyl/alginate blends without ionic crosslinking of alginate
Source: Sci Rep. 2025 Feb 24;15:6609. doi: 10.1038/s41598-025-90389-2 (PMC11850620; doi:10.1038/s41598-025-90389-2)
Supplement: Supplementary file 1 — Supplementary Information. [file 41598_2025_90389_MOESM1_ESM.docx]

# Supplementary

**Bioprinting of mesenchymal stem cells in low concentration Gelatin Methacryloyl /Alginate blends without ionic crosslinking of alginate**

Masoumeh Jahani Kadousaraei^1^, Shuntaro Yamada^1^, Mehmet Serhat Aydin^1^, Ahmad Rashad^1,2^, Noemi Molina Cabeza^3^*,* Samih Mohamed-Ahmed^1^, Cecilie G. Gjerde^1^, Michael Malkoch^3^, Kamal Mustafa^1*^

*Corresponding author: [Kamal.Mustafa@uib.no](mailto:Kamal.Mustafa@uib.no)


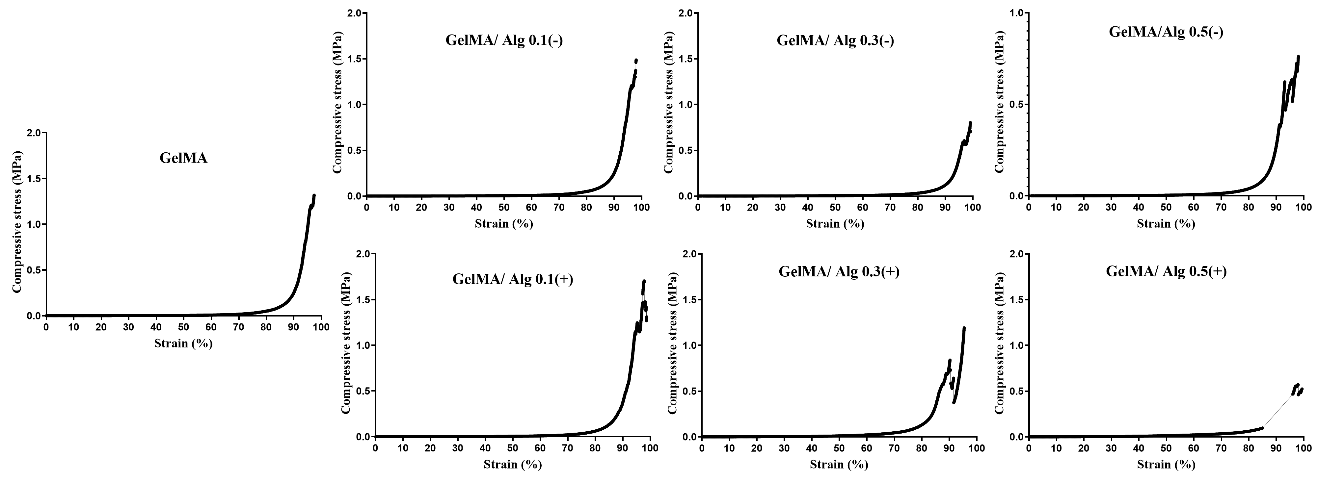


**Figure S1.** **Compressive stress of GelMA/Alg hydrogels with/without ionic crosslinking**

The stress-strain curves of the hydrogels revealed that all hydrogels have elastic behavior until collapsing under high strain ($\sim$90%) (Figure 1S). Non-crosslinked samples showed similar trends to GelMA alone, with a gradual rise in stress at high strains. Adding alginate generally increased compressive stiffness, with this effect being more pronounced in ionically crosslinked samples. Crosslinked hydrogels consistently showed higher stress-handling capacity, starting at lower strain levels, due to improved network stability (at 85% strain for GelMA/Alg 0.3(+) and 0.5(+)). Higher alginate concentrations (0.3% and 0.5%) provided greater elasticity but may reduce mechanical integrity if not crosslinked. Non-crosslinked samples showed delayed stress increases compared to the crosslinked counterparts, highlighting the importance of ionic bonding in reinforcing the hydrogel matrix.
